# Comparing Oncological Outcomes and Surgical Complications of Hand-Assisted, Laparoscopic and Robotic Nephroureterectomy for Upper Tract Urothelial Carcinoma

**DOI:** 10.3389/fonc.2021.731460

**Published:** 2021-10-04

**Authors:** Ching-Chia Li, Chao-Hsiang Chang, Chi-Ping Huang, Jian-Hua Hong, Chao-Yuan Huang, I-Hsuan Alan Chen, Jen-Tai Lin, Chi-Wen Lo, Chih-Chin Yu, Jen-Shu Tseng, Wun-Rong Lin, Wei-Che Wu, Shiu-Dong Chung, Thomas Y. Hsueh, Allen W. Chiu, Yung-Tai Chen, Shin-Hong Chen, Yuan-Hong Jiang, Yao-Chou Tsai, Bing-Juin Chiang, Wei Yu Lin, Yeong-Chin Jou, Chia-Chang Wu, Hsiang-Ying Lee, Hsin-Chih Yeh

**Affiliations:** ^1^ Department of Urology, Kaohsiung Medical University Hospital, Kaohsiung, Taiwan; ^2^ Department of Urology, School of Medicine, College of Medicine, Kaohsiung Medical University, Kaohsiung, Taiwan; ^3^ Graduate Institute of Medicine, College of Medicine, Kaohsiung Medical University, Kaohsiung, Taiwan; ^4^ Department of Urology, China Medical University and Hospital, Taichung, Taiwan; ^5^ School of Medicine, China Medical University, Taichung, Taiwan; ^6^ Department of Urology, National Taiwan University Hospital, College of Medicine, National Taiwan University, Taipei, Taiwan; ^7^ Institute of Biomedical Engineering, National Taiwan University, Taipei, Taiwan; ^8^ Division of Urology, Department of Surgery, Kaohsiung Veterans General Hospital, Kaohsiung, Taiwan; ^9^ Division of Urology, Department of Surgery, Taipei Tzu Chi Hospital, The Buddhist Medical Foundation, New Taipei City, Taiwan; ^10^ School of Medicine, Buddhist Tzu Chi University, Hualien, Taiwan; ^11^ Department of Urology, MacKay Memorial Hospital, Taipei, Taiwan; ^12^ Department of Urology, Mackay Medical College, New Taipei City, Taiwan; ^13^ Institute of Biomedical Informatics, National Yang Ming Chiao Tung University, Taipei, Taiwan; ^14^ Division of Urology, Department of Surgery, Far Eastern Memorial Hospital, New Taipei City, Taiwan; ^15^ Graduate Program in Biomedical Informatics, College of Informatics, Yuan-Ze University, Chung-Li, Taiwan; ^16^ Division of Urology, Department of Surgery, Taipei City Hospital Renai Branch, Taipei, Taiwan; ^17^ Department of Urology, School of Medicine, National Yang Ming Chiao Tung University, Taipei, Taiwan; ^18^ College of Medicine, National Yang Ming Chiao Tung University, Taipei, Taiwan; ^19^ Department of Urology, Taiwan Adventist Hospital, Taipei, Taiwan; ^20^ Department of Urology, Hualien Tzu Chi Hospital, Buddhist Tzu Chi Medical Foundation and Tzu Chi University, Hualien, Taiwan; ^21^ Department of Urology, School of Medicine, College of Medicine, Taipei Medical University, Taipei, Taiwan; ^22^ Department of Urology, Taipei Medical University Hospital, Taipei, Taiwan; ^23^ College of Medicine, Fu-Jen Catholic University, New Taipei City, Taiwan; ^24^ Department of Urology, Cardinal Tien Hospital, New Taipei City, Taiwan; ^25^ Department of Life Science, College of Science, National Taiwan Normal University, Taipei, Taiwan; ^26^ Division of Urology, Department of Surgery, Chang Gung Memorial Hospital, Chiayi, Taiwan; ^27^ Chang Gung University of Science and Technology, Chiayi, Taiwan; ^28^ Department of Medicine, College of Medicine, Chang Gung University, Taoyuan, Taiwan; ^29^ Department of Urology, Ditmanson Medical Foundation Chiayi Christian Hospital, Chiayi, Taiwan; ^30^ Department of Health and Nutrition Biotechnology, Asian University, Taichung, Taiwan; ^31^ Department of Urology, Shuang Ho Hospital, Taipei Medical University, New Taipei City, Taiwan; ^32^ TMU Research Center of Urology and Kidney (TMU-RCUK), Taipei Medical University, Taipei, Taiwan; ^33^ Department of Urology, Kaohsiung Municipal Ta-Tung Hospital, Kaohsiung, Taiwan; ^34^ Graduate Institute of Clinical Medicine, College of Medicine, Kaohsiung Medical University, Kaohsiung, Taiwan

**Keywords:** laparoscopic, robotic, nephroureterectomy, upper tract urothelial carcinoma, hand-assisted

## Abstract

**Purpose:**

This study aimed to compare the oncological outcomes and surgical complications of patients with upper tract urothelial carcinoma (UTUC) treated with different minimally invasive techniques for nephroureterectomy.

**Methods:**

From the updated data of the Taiwan UTUC Collaboration Group, a total of 3,333 UTUC patients were identified. After excluding ineligible cases, we retrospectively included 1,340 patients from 15 institutions who received hand-assisted laparoscopic nephroureterectomy (HALNU), laparoscopic nephroureterectomy (LNU) or robotic nephroureterectomy (RNU) between 2001 and 2021. Kaplan-Meier estimator and Cox proportional hazards model were used to analyze the survival outcomes, and binary logistic regression model was selected to compare the risks of postoperative complications of different surgical approaches.

**Results:**

Among the enrolled patients, 741, 458 and 141 patients received HALNU, LNU and RNU, respectively. Compared with RNU (41.1%) and LNU (32.5%), the rate of lymph node dissection in HALNU was the lowest (17.4%). In both Kaplan-Meier and univariate analysis, the type of surgery was significantly associated with overall and cancer-specific survival. The statistical significance of surgical methods on survival outcomes remained in multivariate analysis, where patients undergoing HALNU appeared to have the worst overall (p = 0.007) and cancer-specific (p = 0.047) survival rates among the three groups. In all analyses, the surgical approach was not related to bladder recurrence. In addition, HALNU was significantly associated with longer hospital stay (p = 0.002), and had the highest risk of major Clavien-Dindo complications (p = 0.011), paralytic ileus (p = 0.012), and postoperative end-stage renal disease (p <0.001).

**Conclusions:**

Minimally invasive surgery can be safe and feasible. We proved that compared with the HALNU group, the LNU and RNU groups have better survival rates and fewer surgical complications. It is crucial to uphold strict oncological principles with sophisticated technique to improve outcomes. Further prospective studies are needed to validate our findings.

## Introduction

Upper tract urothelial carcinoma (UTUC) is a relatively rare malignant tumor, accounting for 5–10% of urothelial carcinoma (1). However, it is reported that the incidence of UTUC in Taiwan is as high as 30–40% (2). UTUC shows more aggressive features than bladder cancer, and more than 60% of patients have invasive disease at the time of diagnosis (3). Radical nephroureterectomy (NU) with bladder cuff excision is still recognized as the gold standard treatment for nonmetastatic UTUC (1). After decades of development, minimally invasive surgeries (MIS) including hand-assisted laparoscopic NU (HALNU), laparoscopic NU (LNU) and robotic NU (RNU) have been introduced as an alternative to open NU (ONU) and widely accepted for the treatment of UTUC.

It is well known that compared with open surgery, the benefits of MIS on perioperative outcomes include lower estimated blood loss, lower blood transfusion rate, shorter hospital stay, less pain, fewer wound complications, and shorter recovery time (4, 5). The oncological outcomes of MIS for UTUC have been controversial, but most previous studies have shown that the survival rate is not inferior compared with open method, especially for organ-confined UTUC (6, 7). HALNU is a combination of laparoscopy and a hand port, through which the hand is inserted into the space created by carbon dioxide insufflation and the specimen is retrieved. Compared with other MIS, HALNU is considered to have lower surgical difficulty and similar oncological results, so it is commonly used. The use of robotic systems is also increasing because it is less technically demanding than pure laparoscopy. For experienced surgeons, all MIS approaches are feasible to maintain basic oncological principles. The intramural ureter and surgical specimen must be completely removed as a whole to prevent tumor residue and spillage (8).

Due to its advantages in the perioperative period and advances in laparoscopic devices (9), MIS is being increasingly used as the current standard in many medical centers around the world. However, reports on the oncological efficacy of different MIS have mixed results. In addition, many previous studies included only a limited number of cases for analysis, and a sizable cohort often failed to comprehensively adjust for potential confounding factors or assess cancer-specific outcomes and bladder recurrence (10–12). The purpose of this study is to analyze a large-scale retrospective cohort derived from multiple institutions in Taiwan to evaluate the oncological outcomes and postoperative complications of UTUC patients treated with minimally invasive NU (miNU).

## Materials and Methods

### Patient Collection

This study was approved by our institutional review board [KMUHIRB-E(I)-20180214]. We retrospectively reviewed the updated data of 15 participating hospitals under the Taiwan UTUC Collaboration Group, and a total of 3,333 UTUC patients were identified. After excluding patients undergoing nephron-sparing surgery (n = 448), ONU (n = 1,230), or patients lacking any parameters of interest (n = 315), we finally included 1,340 patients who received miNU between July 2001 and February 2021. The transperitoneal or retroperitoneal approach of miNU was based on the surgeon discretion. According to different MIS techniques, patients were divided into HALNU, LNU and RNU groups. HALNU was performed by combining hand manipulation through hand port and laparoscopic technique. LNU and RNU only used laparoscopic and robotic instruments to perform surgeries.

In addition to the type of miNU, we collected various parameters for analysis, including age, gender, chronic kidney disease (CKD) stage, bladder cancer history, preoperative hydronephrosis, tumor location, tumor size, tumor focality, bladder cuff status and important pathological features, such as cytology, tumor grade, pathological T stage, lymph node involvement, histological variant, concomitant carcinoma *in situ* (CIS), lymphovascular invasion (LVI) and tumor necrosis. The grade of postoperative complications and the length of hospital stay were also recorded for comparison.

### Definitions and Endpoints

The specimens obtained from miNU were examined by genitourinary pathologists using the same criteria. The pathological staging was based on the 2010 TNM (tumor, lymph node, metastasis) system, and the tumor grade was defined according to the 2004 World Health Organization/International Society of Urologic Pathology consensus classification. The postoperative complications were graded by Clavien-Dindo classification. The regular follow-up program strictly follows the standard guidelines. The endpoint was to compare the oncological outcomes between HALNU, LNU and RNU, including overall survival (OS), cancer-specific survival (CSS), and bladder recurrence-free survival (BRFS). The cause of death was determined by the attending doctor or death certificate. The probability of high-grade surgical complications, paralytic ileus, and postoperative end-stage renal disease (ESRD) among the groups was also analyzed.

### Statistical Analysis

We used one-way ANOVA (analysis of variance) and Pearson’s chi-square test for continuous and categorical variables to compare differences between groups. The Kaplan-Meier estimator was used to estimate the survival function from time-to-event data, and different survival curves were compared using the log-rank test. The Cox proportional hazards model was selected to evaluate the impact of surgical approaches on the prognosis, without or with correction for confounding factors. A binary logistic regression model was used to compare the risks of postoperative complications in the three groups. We used IBM SPSS Statistics software version 26 for analysis. All statistical analyses were two-tailed, and p <0.05 was considered significant. Variables showing statistical significance were included in the adjustment for multivariate analysis.

## Results

In **Table 1**, we compared the clinical and pathological characteristics of patients receiving NU with three different MIS techniques. There were 741, 458 and 141 cases in the HALNU, LNU and RNU groups, respectively. In cytology (p = 0.023), hydronephrosis (p <0.001), tumor location (p <0.001), tumor grade (p = 0.015), pathological N stage (p <0.001), histological variant (p = 0.001), and CIS (p <0.001), there were significant differences between the three groups. Of note, the HALNU group had the lowest rate of lymphadenectomy (17.4%).

**Table 1:** Clinicopathological data of UTUC patients receiving minimally invasive nephroureterectomy.

Variables	Hand-assisted (n = 741)		Pure laparoscopic (n = 458)		Robot-assisted (n = 141)		*p* value^a^
	n	%	n	%	n	%	
Age							0.140
<70 years	349	(47.1)	236	(51.5)	77	(54.6)	
≥70 years	392	(52.9)	222	(48.5)	64	(45.4)	
Gender							0.916
Female	418	(56.4)	264	(57.6)	80	(56.7)	
Male	323	(43.6)	194	(42.4)	61	(43.3)	
Chronic kidney disease							0.561
Stage 1 (≥ 90 ml/min/1.73 m^2^)	47	(6.3)	36	(7.9)	11	(7.8)	
Stage 2 (60-89 ml/min/1.73 m^2^)	226	(30.5)	123	(26.9)	47	(33.3)	
Stage 3 (30-59 ml/min/1.73 m^2^)	309	(41.7)	193	(42.1)	57	(40.4)	
Stage 4 (15-30 ml/min/1.73 m^2^)	51	(6.9)	43	(9.4)	11	(7.8)	
Stage 5 (< 15 ml/min/1.73 m^2^)	108	(14.6)	63	(13.8)	15	(10.6)	
Cytology							0.023*
Negative	479	(64.6)	330	(72.1)	91	(64.5)	
Positive	262	(35.4)	128	(27.9)	50	(35.5)	
History of bladder cancer							0.524
No	578	(78.0)	361	(78.8)	116	(82.3)	
Yes	163	(22.0)	97	(21.2)	25	(17.7)	
Hydronephrosis							<0.001**
No	244	(32.9)	208	(45.4)	80	(56.7)	
Yes	497	(67.1)	250	(54.6)	61	(43.3)	
Tumor location							<0.001**
Renal pelvis	369	(49.8)	223	(48.7)	83	(59.3)	
Ureter	224	(30.2)	176	(38.4)	33	(23.6)	
Synchronous	148	(20.0)	59	(12.9)	24	(17.1)	
Tumor size							0.685
<3 cm	208	(28.1)	139	(30.3)	42	(29.8)	
≥3 cm	533	(71.9)	319	(69.7)	99	(70.2)	
Multifocality							0.095
No	479	(64.6)	323	(70.5)	91	(64.5)	
Yes	262	(35.4)	135	(29.5)	50	(35.5)	
Tumor grade							0.015*
Low grade	144	(19.4)	63	(13.8)	18	(12.8)	
High grade	597	(80.6)	395	(86.2)	123	(87.2)	
Pathological T stage							0.906
pTis/pTa	147	(19.8)	82	(17.9)	27	(19.1)	
pT1	182	(24.6)	122	(26.6)	37	(26.2)	
pT2	148	(20.0)	83	(18.1)	23	(16.3)	
pT3	239	(32.3)	156	(34.1)	47	(33.3)	
pT4	25	(3.4)	15	(3.3)	7	(5.0)	
Pathological N stage							<0.001**
pN0	110	(14.8)	120	(26.2)	48	(34.0)	
pNx	612	(82.6)	309	(67.5)	83	(58.9)	
pN+	19	(2.6)	29	(6.3)	10	(7.1)	
Histological variant							0.001**
No	689	(93.0)	396	(86.5)	128	(90.8)	
Yes	52	(7.0)	62	(13.5)	13	(9.2)	
Carcinoma *in situ*							<0.001**
No	663	(89.5)	354	(77.3)	109	(77.3)	
Yes	78	(10.5)	104	(22.7)	32	(22.7)	
Lymphovascular invasion							0.154
No	565	(76.2)	369	(80.6)	114	(80.9)	
Yes	176	(23.8)	89	(19.4)	27	(19.1)	
Tumor necrosis							0.230
No	603	(81.4)	385	(84.1)	122	(86.5)	
Yes	138	(18.6)	73	(15.9)	19	(13.5)	
Residual bladder cuff							0.711
No	628	(84.8)	380	(83.0)	119	(84.4)	
Yes	113	(15.2)	78	(17.0)	22	(15.6)	
Major complication (Clavien-Dindo ≥ III)							0.033*
No	686	(92.6)	437	(95.4)	137	(97.2)	
Yes	55	(7.4)	21	(4.6)	4	(2.8)	
Paralytic ileus							0.034
No	710	(95.8)	448	(97.8)	140	(99.3)	
Yes	31	(4.2)	10	(2.2)	1	(0.7)	
Postoperative ESRD							0.001**
No	635	(85.7)	420	(91.7)	133	(94.3)	
Yes	106	(14.3)	38	(8.3)	8	(5.7)	
Hospital stay (Mean ± SD), days^b^	9.61 ± 6.95		8.97 ± 4.96		7.73 ± 3.35		0.002**

^a^Chi-square test calculated for the difference in variables. ^b^One-way analysis of variance calculated for the difference among means. ESRD, end-stage renal disease. *< 0.05, **< 0.01.

### Univariate Survival Analysis

**Figure 1:**
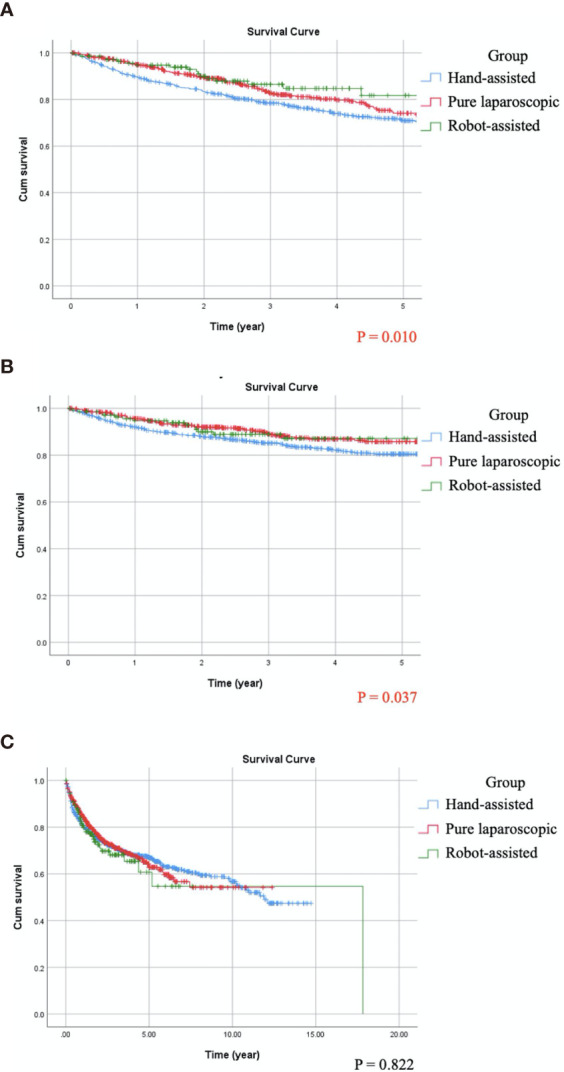
Compare Kaplan-Meier curves between patients receiving different minimally invasive nephroureterectomy by log-rank test. **(A)** Overall survival, p = 0.010. **(B)** Cancer-specific survival, p = 0.037. **(C)** Bladder recurrence-free survival, p = 0.822.

Kaplan-Meier analysis showed that OS (p = 0.010) and CSS (p = 0.037) were significantly different between the three miNU groups (**Figures 1A, B**). The 5-year OS and CSS rates of HALNU were 71% and 80%, respectively, LNU were 74% and 86%, and RNU were 82% and 87%. In univariate analysis, the significant factors for both OS and CSS were age, CKD, history of bladder cancer, hydronephrosis, pathological T and N stages, histologic variant, LVI, tumor necrosis and types of surgery (**Table 2**, all p <0.05). As for BRFS, there was no statistical difference between the groups (**Figure 1C**, p = 0.822). Gender, bladder cancer history, tumor location, tumor focality, histologic variant, and CIS were significantly associated with bladder recurrence (**Table 2**, all p <0.05).

**Table 2:** Comparative univariate survival analysis of UTUC patients receiving minimally invasive NU.

Univariate analysis			OS		CSS		BRFS	
			HR (95% CI)	*p* value	HR (95% CI)	*p* value	HR (95% CI)	*p* value
Age				<0.001**		0.002**		0.757
<70 years			1		1		1	
≥70 years			2.134 (1.738, 2.619)		1.536 (1.166, 2.024)		1.033 (0.841, 1.268)	
Gender				0.331		0.613		<0.001**
Female			1		1		1	
Male			1.103 (0.905, 1.344)		1.073 (0.817, 1.140)		1.837 (1.495, 2.257)	
Chronic kidney disease				<0.001**		0.021*		0.302
Stage 1 (≥ 90 ml/min/1.73 m^2^)			1		1		1	
Stage 2 (60-89 ml/min/1.73 m^2^)			1.162 (0.681, 1.982)	0.583	1.213 (0.617, 2.388)	0.576	1.048 (0.679, 1.616)	0.833
Stage 3 (30-59 ml/min/1.73 m^2^)			1.907 (1.144, 3.181)	0.013*	1.702 (0.887, 3.265)	0.110	1.021 (0.668, 1.560)	0.923
Stage 4 (15-30 ml/min/1.73 m^2^)			2.291 (1.277, 4.110)	0.005**	1.837 (0.848, 3.981)	0.123	1.348 (0.799, 2.275)	0.263
Stage 5 (< 15 ml/min/1.73 m^2^)			2.589 (1.511, 4.437)	0.001**	1.786 (0.878, 3.633)	0.109	1.172 (0.726, 1.892)	0.516
Cytology				0.913		0.032*		0.864
Negative			1		1		1	
Positive			1.012 (0.821, 1.247)		1.354 (1.026, 1.787)		0.981 (0.790, 1.219)	
History of bladder cancer				0.001**		<0.001**		<0.001**
No			1		1		1	
Yes			1.482 (1.186, 1.851)		1.909 (1.427, 2.554)		1.802 (1.443, 2.252)	
Hydronephrosis				<0.001**		<0.001**		0.092
No			1		1		1	
Yes			1.589 (1.277, 1.976)		1.722 (1.270, 2.336)		1.200 (0.971, 1.484)	
Tumor location				0.009**		<0.001**		<0.001**
Renal pelvis			1		1		1	
Ureter			1.199 (0.960, 1.497)	0.109	1.342 (0.979, 1.838)	0.067	1.186 (0.940, 1.498)	0.151
Synchronous			1.403 (1.077, 1.829)	0.012*	1.974 (1.397, 2.790)	<0.001**	1.693 (1.295, 2.213)	<0.001**
Tumor size				0.001**		<0.001**		0.760
<3 cm			1		1		1	
≥3 cm			1.489 (1.183, 1.874)		2.479 (1.708, 3.596)		1.035 (0.830, 1.290)	
Multifocality				0.002**		<0.001**		<0.001**
No			1		1		1	
Yes			1.381 (1.127, 1.693)		1.863 (1.418, 2.448)		1.592 (1.291, 1.962)	
Tumor grade				0.001**		<0.001**		0.223
Low grade		1			1		1	
High grade			1.602 (1.204, 2.132)		3.292 (1.913, 5.665)		0.856 (0.666, 1.099)	
Pathological T stage				<0.001**		<0.001**		0.774
pTis/pTa		1			1		1	
pT1			1.213 (0.850, 1.731)	0.286	1.172 (0.583, 2.356)	0.656	1.047 (0.778, 1.409)	0.763
pT2			1.674 (1.166, 2.403)	0.005**	3.011 (1.596, 5.678)	0.001**	1.093 (0.797, 1.499)	0.581
pT3			2.866 (2.087, 3.936)	<0.001**	6.941 (3.912, 12.316)	<0.001**	1.068 (0.799, 1.430)	0.656
pT4			7.162 (4.543, 11.290)	<0.001**	16.067 (7.975, 32.369)	<0.001**	0.311 (0.098, 0.987)	0.047*
Pathological N stage				0.003*		<0.001**		0.867
pN0		1			1		1	
pNx			1.097 (0.836, 1.440)	0.502	1.141 (0.779, 1.670)	0.499	0.978 (0.755, 1.267)	0.866
pN+			3.079 (1.959, 4.838)	<0.001**	4.405 (2.557, 7.589)	<0.001**	0.971 (0.527, 1.789)	0.924
Histological variant				<0.001**		<0.001**		0.032*
No		1			1		1	
Yes			2.004 (1.496, 2.685)		2.743 (1.928, 3.902)		0.611 (0.389, 0.958)	
Carcinoma *in situ*				0.201		0.210		0.008**
No		1			1		1	
Yes			1.188 (0.912, 1.549)		1.252 (0.881, 1.781)		1.419 (1.093, 1.842)	
Lymphovascular invasion				<0.001**		<0.001**		0.434
No		1			1		1	
Yes			2.232 (1.804, 2.761)		3.396 (2.579, 4.472)		1.108 (0.858, 1.430)	
Tumor Necrosis				<0.001**		<0.001**		0.985
No		1			1		1	
Yes			1.535 (1.211, 1.946)		1.813 (1.327, 2.476)		1.003 (0.763, 1.317)	
Residual bladder cuff				0.582		0.361		0.728
No		1			1		1	
Yes			0.926 (0.703, 1.218)		0.833 (0.563, 1.233)		1.049 (0.800, 1.376)	
Type of minimally invasive NU				0.002*		0.019*		0.837
Hand-assisted			1		1		1	
Pure laparoscopic			0.784 (0.619, 0.991)	0.042*	0.678 (0.490, 0.938)	0.019*	0.987 (0.788, 1.237)	0.911
Robot-assisted			0.535 (0.327, 0.876)	0.013*	0.681 (0.392, 1.182)	0.172	1.070 (0.744, 1.539)	0.715

CI, confidence interval; HR, hazard ratio; OS, overall survival; CSS, cancer-specific survival; BRFS, bladder recurrence-free survival; NU, nephroureterectomy. *< 0.05, **< 0.01.

### Multivariate Survival Analysis

As shown in **Table 3**, CKD was a prognostic factor of OS (p <0.001) rather than CSS, while tumor size (p = 0.009) and focality (p = 0.043) were significant for CSS but not for OS. The independent significant parameters for both OS and CSS were age (p <0.001; p = 0.008), history of bladder cancer (p = 0.001; p <0.001), hydronephrosis (p = 0.005; p = 0.010), pathological T stage (p <0.001; p <0.001), pathological N stage (p = 0.031; p = 0.006), histological variant (p = 0.030; p = 0.004), LVI (p = 0.028; p = 0.017), and type of surgery (p = 0.007; p = 0.047). Specifically, after adjusting for various confounding factors, patients receiving HALNU had the worst OS and CSS. In multivariate analysis, different MIS approaches had no effect on bladder recurrence (p = 0.829). Instead, gender (p <0.001), bladder cancer history (p = 0.002), histologic variant (p = 0.038), and CIS (p = 0.026) were significantly correlated with BRFS.

**Table 3:** Comparative multivariate survival analysis of UTUC patients receiving minimally invasive NU.

Multivariate analysis	OS		CSS		BRFS	
	HR (95% CI)	*p* value	HR (95% CI)	*p* value	HR (95% CI)	*p* value
Age		<0.001**		0.008**		
<70 years	1		1			
≥70 years	2.187 (1.762, 2.715)		1.478 (1.106, 1.977)			
Gender						<0.001**
Female					1	
Male					1.763 (0.431, 2.173)	
Chronic kidney disease		<0.001**		0.336		
Stage 1 (≥ 90 ml/min/1.73 m^2^)	1		1			
Stage 2 (60-89 ml/min/1.73 m^2^)	0.867 (0.504, 1.492)	0.607	0.993 (0.496, 1.988)	0.985		
Stage 3 (30-59 ml/min/1.73 m^2^)	1.168 (0.691, 1.972)	0.562	1.122 (0.572, 2.201)	0.739		
Stage 4 (15-30 ml/min/1.73 m^2^)	1.403 (0.773, 2.548)	0.265	0.991 (0.448, 2.192)	0.982		
Stage 5 (< 15 ml/min/1.73 m^2^)	1.966 (1.136, 3.404)	0.016*	1.259 (0.607, 2.612)	0.537		
Cytology				0.109		
Negative			1			
Positive			1.268 (0.948, 1.697)			
History of bladder cancer		0.001**		<0.001**		0.002**
No	1		1		1	
Yes	1.479 (1.163, 1.880)		1.842 (1.333, 2.545)		1.459 (1.150, 1.852)	
Hydronephrosis		0.005**		0.010**		
No	1		1			
Yes	1.415 (1.112, 1.801)		1.578 (1.117, 2.228)			
Tumor location		0.309		0.990		0.052
Renal pelvis	1		1		1	
Ureter	1.057 (0.822, 1.358)	0.666	1.279 (0.889, 1.839)	0.185	1.203 (0.951, 1.527)	0.122
Synchronous	0.797 (0.558, 1.137)	0.211	0.961 (0.603, 1.530)	0.867	1.334 (0.949, 1.874)	0.097
Tumor size		0.260		0.009**		
<3 cm	1		1			
≥3 cm	1.158 (0.897, 1.494)		1.700 (1.140, 2.535)			
Multifocality		0.100		0.043*		0.235
No	1		1		1	
Yes	1.263 (0.956, 1.668)		1.476 (1.013, 2.151)		1.187 (0.895, 1.576)	
Tumor grade		0.851		0.201		
Low grade	1		1			
High grade	0.970 (0.709, 1.328)		1.465 (0.815, 2.632)			
Pathological T stage		<0.001**		<0.001**		
pTis/pTa	1		1			
pT1	1.309 (0.904, 1.896)	0.154	0.964 (0.469, 1.984)	0.896		
pT2	1.548 (1.042, 2.298)	0.030*	1.933 (0.984, 3.799)	0.056		
pT3	2.588 (1.772, 3.781)	<0.001**	3.909 (2.054, 7.439)	<0.001**		
pT4	6.104 (3.589, 10.380)	<0.001**	7.524 (3.387, 16.712)	<0.001**		
Pathological N stage		0.031*		0.006**		
pN0	1		1			
pNx	1.140 (0.861, 1.510)	0.359	1.300 (0.875, 1.932)	0.194		
pN+	1.945 (1.215, 3.116)	0.006**	2.407 (1.367, 4.237)	0.002**		
Histological variant		0.030*		0.004**		0.038*
No	1		1		1	
Yes	1.423 (1.035, 1.957)		1.762 (1.193, 2.602)		0.617 (0.390, 0.974)	
Carcinoma *in situ*						0.026*
No					1	
Yes					1.355 (1.037, 1.772	
Lymphovascular invasion		0.028*		0.017*		
No	1		1			
Yes	1.310 (1.029, 1.667)		1.454 (1.068, 1.978)			
Tumor Necrosis		0.865		0.882		
No	1		1			
Yes	0.978 (0.757, 1.263)		0.974 (0.692, 1.371)			
Type of minimally invasive NU		0.007**		0.047*		0.829
Hand-assisted	1		1		1	
Pure laparoscopic	0.768 (0.599, 0.986)	0.038*	0.663 (0.467, 0.940)	0.021*	0.978 (0.776, 1.232)	0.847
Robot-assisted	0.534 (0.318, 0.896)	0.018*	0.730 (0.413, 1.290)	0.279	1.082 (0.751, 1.558)	0.673

CI, confidence interval; HR, hazard ratio; OS, overall survival; CSS, cancer-specific survival; BRFS, bladder recurrence-free survival; NU, nephroureterectomy. *< 0.05, **< 0.01.

### Postoperative Complications

There were significant differences in postoperative complications and hospital stay according to the MIS methods (**Table 1**). HALNU was significantly associated with higher Clavien-Dindo complications (p = 0.033), more paralytic ileus (p = 0.034), more postoperative ESRD (p = 0.001), and longer hospital stay (p = 0.002). Through logistic regression (**Table 4**), the HALNU group also had the highest risk of major complications (p = 0.011), ileus (p = 0.012), and ESRD (p <0.001). After correcting the confounders that can affect postoperative renal function, namely CKD and hydronephrosis, the HALNU approach still had a significantly higher chance of ESRD than the LNU or RNU method (p <0.001).

**Table 4:** Comparative univariate/multivariate logistic regression analysis for postoperative complications.

Variables	Clavien-Dindo ≥ III^c^		Paralytic ileus^c^		Postoperative ESRD^c^		Postoperative ESRD^d^	
	OR (95% CI)	*p* value	OR (95% CI)	*p* value	OR (95% CI)	*p* value	OR (95% CI)	*p* value
Type of minimally invasive NU		0.011*		0.012*		<0.001**		<0.001**
Hand-assisted	1		1		1		1	
Pure laparoscopic	0.599 (0.357, 1.005)	0.052	0.511 (0.248, 1.053)	0.069	0.542 (0.367, 0.801)	0.002**	0.533 (0.357, 0.794)	0.002**
Robot-assisted	0.364 (0.130, 1.022)	0.055	0.164 (0.022, 1.208)	0.076	0.360 (0.171, 0.757)	0.007**	0.390 (0.183, 0.830)	0.015*
Chronic kidney disease						<0.001**		<0.001**
Stage 1 (≥ 90 ml/min/1.73 m^2^)					1		1	
Stage 2 (60-89 ml/min/1.73 m^2^)					0.798 (0.287, 2.222)	0.666	0.758 (0.271, 2.116)	0.596
Stage 3 (30-59 ml/min/1.73 m^2^)					3.060 (1.206, 7.762)	0.019*	2.896 (1.136, 7.383)	0.026*
Stage 4-5 (< 30 ml/min/1.73 m^2^)					3.516 (1.356, 9.114)	0.010**	3.339 (1.281, 8.701)	0.014*
Hydronephrosis						0.019*		0.319
No					1		1	
Yes					1.541 (1.072, 2.216)		1.211 (0.831, 1.765)	

^c^Univariate logistic regression analysis. ^d^Multivariate logistic regression analysis. ESRD, end-stage renal disease; CI, confidence interval; OR, odds ratio; NU, nephroureterectomy. *<0.05, **< 0.01.

## Discussion

ONU with bladder cuff excision is the standard of treatment for UTUC. With the development of new surgical techniques, miNU has become a popular method. However, the efficacy and safety of MIS approach for locally advanced UTUC has been a concern. In the latest version of guideline, T3/T4 and/or node-positive tumors are contraindications for LNU (1). The study of Shigeta et al. conducted a subgroup analysis and the results showed that CSS and BRFS rates of the LNU group in T3 patients were lower than those of the ONU group (13). Similarly, Kim et al. demonstrated that in pT3/T4 patients, the 5-year OS and CSS rates in the LNU group were lower than those in the ONU group (14). Therefore, concerns about compromised oncological integrity may prevent surgeons from choosing MIS for these patients. Nevertheless, several studies have reported comparable oncological outcomes for open and MIS approaches (6, 15–17), which are believed to depend on following rigorous oncological principles.

The results of our multi-institution series indicated that both LNU and RNU had better survival rates than HALNU in multivariate analysis, and underlying plausible reasons were hypothesized. The first assumption is that the quality of lymph node dissection (LND), which can be affected by surgical methods and lead to prognostic variations. In patients undergoing radical prostatectomy for prostate cancer, a large difference in pelvic LND between open and MIS approaches (83.1% *vs* 16.9%) was reported (18). Although the benefits of conventional lymphadenectomy for UTUC were controversial, a meta-analysis concluded that LND may prolong CSS in patients with muscle-invasive disease (19). This concept was supported by previous studies. Abe et al. showed that when performing regional LND, the survival outcome between LNU and ONU was equivalent (20). In the study of Kim et al. (14), the worse OS and CSS in the LNU group may be attributed to the lower LND rate (13.0%) compared with the ONU group (21.0%). In addition, previous studies demonstrated that LND was more likely to be performed with robot assistance, resulting in higher lymph node yield than other MIS (11, 7 and 5 nodes were obtained with RNU, LNU and HALNU, respectively) (7, 10). In our study, a similar trend was found in the proportion of lymphadenectomy with different miNU (RNU: 41.1%, LNU: 32.5%, HALNU: 17.4%; p <0.001), and the RNU group did have the highest LND rate. In a large population cohort of 16,619 UTUCs, 15.4% of cases underwent LND (10). It can be inferred that although the proportion of LND in our HALNU group is not low, meticulous LND in the LNU and RNU groups may translate into better survival outcomes.

The probability of carrying out LND in different MIS may also be related to the surgeon’s experience. When performing minimally invasive radical prostatectomy, Prasad et al. proved that high-volume surgeons were more likely to execute pelvic LND than low-volume surgeons (27.7% *vs* 5.7%) (18). In this cohort, the enrolled cases spanned 20 years, and therefore multiple surgeons were included. LNU and RNU were generally performed by experienced hands who can accomplish LND proficiently. On the contrary, most surgeons can perform HALNU with ease, but a considerable number of inexperienced surgeons may be responsible for a relatively lower LND rate, leading to a worse prognosis.

For tumor extraction, an endobag will be used to retrieve the specimen during LNU and RNU. However, when performing HALNU, the specimen will not be placed in an isolation bag before it is removed by hand through the hand port. Therefore, HALNU may have a higher risk of inadvertent tumor contamination or spillage into adjacent tissues. An article from Japan indicated that the recurrence-free survival of HALNU was significantly lower than that of LNU (21). In addition, the higher incidence of high-grade complications in the HALNU group may be related to poor survival. Lastly, patients undergoing HALNU had significantly worse renal function during the postoperative follow-up period compared with the LNU or RNU groups. Impaired renal function was associated with an increase in cardiovascular events (22) and hindered the use of cisplatin-based adjuvant chemotherapy, both of which caused a worse prognosis. Based on these assumptions, our results indicated that LNU and RNU can achieve better survival outcomes than HALNU.

Postoperative systemic therapies may affect clinical outcomes. In the HALNU, LNU, and RNU groups, 165 (22.3%), 112 (24.5%), and 34 (24.1%) patients received chemotherapy after surgery, and 7 (0.9%), 3 (0.7%), and 1 (0.7%) patients received postoperative immunotherapy. There was no statistical difference in systemic treatment between the three groups (p = 0.894). We attempted to incorporate this factor for analysis and found that the use of systemic therapy is harmful to survival outcomes, which is obviously unreasonable. We suppose the main reason is that we are unable to determine whether these therapies are used for adjuvant, salvage or palliative purpose. Therefore, we did not include this parameter in the multivariate analysis.

Compared with ONU, miNU has advantages in perioperative results, such as less bleeding and faster recovery. Among different miNU, our results showed that HALNU had the longest hospital stay, which may be related to more pain caused by a large incision and hand manipulation. Patients receiving HALNU also had more postoperative ileus. If more opioid analgesics are given because of wound pain, gastrointestinal motility may decrease (23). Direct hand contact with the intestines can trigger local inflammation, which in turn impairs bowel movements (24). In addition, the major Clavien-Dindo complications were highest in HALNU and lowest in RNU. Three-dimensional vision, greater flexibility, instrument accuracy, and better ergonomics of RNU may translate into lower complications (25, 26). A meta-analysis of NU techniques has demonstrated that RNU has a low incidence of intraoperative complications (7). In short, it is assumed that more delicate surgical procedures can reduce postoperative complications.

In our study, there was no significant difference in preoperative renal function between the three MIS groups, but patients who received HALNU had a higher incidence of postoperative ESRD. In theory, removal of the diseased kidney will activate the compensatory hyperfiltration and hypertrophy of the other kidney, so the decline in renal function is tolerable if the contralateral kidney is healthy. It has been reported that the estimated glomerular filtration rate of American patients after NU has decreased by an average of 24% (27). However, when comparing the renal outcome of patients with renal cell carcinoma and UTUC in Taiwan after radical surgery, the latter had a significantly higher rate of worsening renal function or ESRD (28). The endemic specific risk factors of UTUC in Taiwan, including arsenic contamination in drinking water and herbs containing aristolochic acid, are potential culprits; a dose-dependent association between the two and CKD has been demonstrated (29, 30). After long-term exposure to these nephrotoxic carcinogens, UTUC can precede renal dysfunction, leading to postoperative renal function deterioration. HALNU was common decades ago, but LNU and RNU gradually became popular after the Taiwan government banned aristolochic acid in 2003. Therefore, the poor renal outcome in the HALNU group may be due to the continuous renal damage caused by carcinogenic nephrotoxin.

Although this study provides important insights for comparing MIS techniques of NU, it does have some limitations. First, it is carried out in retrospective design. Second, RNU is a relatively new approach and is being increasingly implemented, so there are fewer patients in this group and the follow-up period is shorter. Third, despite the large case number, this study involves multiple institutions, spanning 2 decades, and inevitably has a heterogeneous background. Differences in the experience of multiple surgeons may also be a source of bias. Lastly, we do not know the exposure dose of endemic risk factors, so we cannot assess the indolent damage to the kidneys of these carcinogenic nephrotoxins. Nevertheless, this is by far the largest cohort formed by miNU. All previous studies larger than ours came from database analysis and usually lacked key clinical, pathological and oncological information for each patient. As the first and largest cohort to compare the outcomes between HALNU, LNU and RNU, this study was strengthened by comprehensively correcting the effects of confounding covariates.

There is evidence that miNU is safe and feasible to treat UTUC. We demonstrated that compared with LNU and RNU, HALNU has a worse survival rate and more postoperative complications. The quality of LND, surgeon experience, the use of endobag, direct contact with hands and intestines, and exposure to nephrotoxic carcinogens are all possible factors that can explain our findings. It is essential to adhere to the oncological principle with skilled techniques during the operation, and subtle and careful surgery can achieve better outcomes.

## Data Availability Statement

The raw data supporting the conclusions of this article will be made available by the authors, without undue reservation.

## Ethics Statement

The studies involving human participants were reviewed and approved by Kaohsiung Medical University Hospital [KMUHIRB-E(I)-20180214]. Written informed consent for participation was not required for this study in accordance with the national legislation and the institutional requirements.

## Author Contributions

Y-CT, TH, and H-CY conceived the project. All authors collected the data. H-CY analyzed the results. H-YL and C-CL drafted the manuscript. H-CY edited the manuscript. All authors contributed to the article and approved the submitted version.

## Funding

This study was supported by Kaohsiung Municipal Ta-Tung Hospital (kmtth-109-R04) and supported partially by the Ministry of Science and Technology (MOST 109-2314-B-037-095), Kaohsiung Medical University (KMU-KI109002), Kaohsiung Medical University Hospital (KMUH-DK(C)110006), Kaohsiung Medical University Regenerative Medicine and Cell Therapy Research Center (KMU-TC109A02), and Kaohsiung Medical University Center for Liquid Biopsy and Cohort Research (KMU-TC109B05).

## Conflict of Interest

The authors declare that the research was conducted in the absence of any commercial or financial relationships that could be construed as a potential conflict of interest.

## Publisher’s Note

All claims expressed in this article are solely those of the authors and do not necessarily represent those of their affiliated organizations, or those of the publisher, the editors and the reviewers. Any product that may be evaluated in this article, or claim that may be made by its manufacturer, is not guaranteed or endorsed by the publisher.
